# Supplementary material for: T-cell receptor structures and predictive models reveal comparable alpha and beta chain structural diversity despite differing genetic complexity
Source: Commun Biol. 2025 Mar 4;8:362. doi: 10.1038/s42003-025-07708-6 (PMC11880327; doi:10.1038/s42003-025-07708-6)
Supplement: Supplementary file 1 — Supplementary Information [file 42003_2025_7708_MOESM1_ESM.pdf]

Supplementary Information: T-cell receptor structures and predictive models reveal comparable alpha and beta chain structural diversity despite differing genetic complexity

Nele P. Quast, Brennan Abanades, Bora Guloglu, Vijaykumar Karuppiah, Stephen Harper, Matthew I. J. Raybould, Charlotte M. Deane*

Contents

1 SI Tables (1-12) **2**

2 SI Figures (1-22) **6**

3 SI Notes (1-3) **21**

3.1 Accurate prediction of both CDR3α and CDR3β is required for TCR:pMHC interface characterisation using physics-driven docking . . . 21

3.2 Molecular dynamics suggests ensembles of models generated by TCR structure predictors may represent alternative, physically plausible apo states . . . 24

3.3 Structural distance calculation for length-mismatched CDR loops . . . 27

1 SI Tables (1-12)

4nhu	5wlg	6zkx	7dzm	7dzn	7f5k	7fje	7l1d	7n2o	7n2q
7n2r	7n2s	7n5c	7n5p	7na5	7ndq	7ndt	7nme	7ow5	7pb2
7pbc	7pbe	7pdw	7phr	7q9b	7qpj	7r7z	7rdv	7rk7	7rm4
7rrg	7s8i	7sg1	7su9	7t2b	7t2c	7z50	7zt7	8cx4	8d5q
8dnt	8es9	8gvb	8gvi	8shi					

SI Table 1: PDB codes of 45 TCRs in the test set.

Length	CDR1α	CDR2α	CDR3α	CDR1β	CDR2β	CDR3β
5	15	19	-	21	5	-
6	64	33	-	7	57	-
7	17	59	-	-	8	-
8	-	22	8	-	-	-
9	-	-	37	-	-	24
10	-	-	80	-	-	54
11	-	-	121	-	-	99
12	-	-	67	-	-	136
13	-	-	77	-	-	66
Total	96	133	390	28	70	379

SI Table 2: Number of clusters of TCRBuilder2+ training structures by greedy clustering by RMSD with 1Å clustering threshold. '-' indicates less than 10 loops of that length existed in the training set to cluster. There are approximately as many CDR3α clusters as CDR3β clusters, suggesting CDR3α loops are as structurally diverse as CDR3β loops. There are substantially more CDR3 loop clusters than CDR1 and CDR2 clusters.

	A	fwa	CDR1α	CDR2α	CDR3α	B	fwb	CDR1β	CDR2β	CDR3β
TCRBuilder2	1.0000	1.0000	1.0000	1.0000	1.0000	1.0000	1.0000	1.0000	1.0000	1.0000
TCRBuilder2+	0.4907	0.1261	0.9250	0.1720	0.9977	0.9135	0.6431	0.1555	0.3081	0.5788
Alphafold3	**0.0007**	**0.0000**	**0.0435**	0.8349	0.2956	0.5285	0.2293	0.5983	0.3693	0.5021
ABodyBuilder2	**0.0000**	**0.0000**	**0.0000**	**0.0000**	**0.0000**	**0.0000**	**0.0000**	**0.0000**	**0.0000**	**0.0000**
Alphafold Multimer	0.0601	**0.0000**	0.3340	0.3420	0.7982	0.9320	0.5170	0.8258	0.1368	0.4312
LYRA*	0.6174	0.0481	0.2020	0.8099	0.0045	0.7554	0.9723	0.1043	0.4895	0.1154
TCRmodel	0.1099	0.1295	0.0869	0.1310	0.0107	0.6736	0.7640	0.0916	0.7466	0.4053

SI Table 3: P values of a two-tailed test on the t-statistic of the RMSD of a given model compared to TCRBuilder2. Statistically significant values (5% threshold) in bold. Apart from ABodyBuilder2, differences in the RMSD of different models are not statistically significant, with the exception of the α chain framework for Alphafold Multimer, and the α chain framework, CDRα1, and whole α chain for Alphafold3.

Model	A	fwa	CDR1α	CDR2α	CDR3α	B	fwb	CDR1β	CDR2β	CDR3β
TCRBuilder2+	43	45	37	43	21	42	45	44	44	34
TCRBuilder2	45	45	36	45	23	43	45	45	45	26
Alphafold Multimer	44	45	37	43	27	43	45	44	45	32
TCRmodel	35	39	28	37	10	37	39	36	39	23
Alphafold3	45	45	39	43	25	44	45	44	43	33
LYRA*	35	38	27	36	8	38	38	36	38	20
ABodyBuilder2	0	0	0	0	6	0	1	0	0	4

SI Table 4: Number of test set predictions per model with RMSD < 2Å. TCRBuilder2+ outperforms TCRBuilder2 and Alphafold Multimer for CDR3β, but retrieves fewer CDR3α structures. *LYRA, the homology method, could only be evaluated on 38 of the 45 test structures for which a structure was generated.

	0.5	1.0	1.5	2.0	2.5	3.0	3.5	4.0	4.5
CDR1α	501	126	45	23	12	9	4	3	3
CDR1β	215	34	9	6	5	2	1	1	1
CDR2α	604	200	84	42	27	17	9	7	7
CDR2β	415	105	45	23	13	8	5	2	2
CDR3α	994	840	410	148	62	22	13	5	4
CDR3β	1000	828	417	178	71	32	19	11	5

SI Table 5: Number of clusters for each CDR loop identified for a sample of 1000 OTS TRCBuilder2+ structure predictions for varying RMSD thresholds in Ångströms.

	0.5	1.0	1.5	2.0	2.5	3.0	3.5	4.0	4.5
CDRL1	90	31	19	16	11	7	6	5	5
CDRH1	121	19	8	5	2	2	2	1	1
CDRL2	25	9	7	6	5	4	4	3	3
CDRH2	156	36	18	11	9	5	4	4	3
CDRL3	391	131	56	23	12	7	6	4	4
CDRH3	995	880	659	406	208	105	61	39	25

SI Table 6: Number of clusters for each CDR loop identified for a sample of 1000 OAS ABodyBuilder2 structure predictions for varying RMSD thresholds in Ångströms.

Gene type	TRA	TRB	IGL/K	IGH
V	45	48	33/41	57
D	-	2	-	23
J	51	13	5/5	6
Potential combinatorial diversity	2295	1248	165/205	7866

SI Table 7: Number of functional IMGT [1] catalogued human genes for the TRA, TRB, IGL/K, and IGH loci. TRA and TRB are the TCR alpha and beta loci respectively. IGL/K are the antibody light chain loci, split into lambda and kappa genes. IGH is the antibody heavy chain locus. V, J and D are the variable, joining, and diversity segments respectively. A relatively large number of joining genes have been observed for the TRA locus compared to TRB and IG loci. The large number of TRAJ and low number of TRBD result in comparable potential combinatorial diversity of TCR alpha and beta sequences.

Allele type	TRA	TRB	IGL/K	IGH
V	112	121	99/86	346
D	-	3	-	33
J	61	15	7/9	16
Potential combinatorial diversity	6852	5445	693/774	182688

SI Table 8: Number of functional IMGT [1] catalogued human alleles for the TRA, TRB, IGL/K, and IGH loci. TRA and TRB are the TCR alpha and beta loci respectively. IGL/K are the antibody light chain loci, split into lambda and kappa genes. IGH is the antibody heavy chain locus. V, J and D are the variable, joining, and diversity segments respectively.

	1.0	0.9	0.8	0.7	0.6	0.5	0.4	0.3	0.2	0.1	0.0
CDR1α	65	65	50	49	30	22	8	7	2	1	1
CDR2α	59	59	50	48	37	20	16	9	6	2	1
CDR3α	1968	1705	1012	450	168	64	32	15	3	2	1
CDR1β	44	44	44	17	16	8	6	6	2	2	1
CDR2β	57	57	47	47	23	22	11	10	3	2	1
CDR3β	1998	1938	1490	758	256	64	22	10	3	2	1

SI Table 9: Number of sequence clusters emerging from 2000 randomly sampled TCRs from OTS, by TCR loop and sequence identity clustering threshold.

Model	Compute resource	Mean inference time per TCR	Relative speed up
TCRBuilder2+	CPU i7 core	24.4 sec	261 x
Alphafold Multimer	GPU Ampere A100	110 min 19.6 sec	1 x

SI Table 10: Inference time of TCRBuilder2+ compared to Alphafold Multimer. TCRBuilder2+ offers a 261-times speed up on computationally cheaper CPU compared to Alphafold Multimer running on GPU, enabling inference of TCR structures at repertoire scale with lower compute resource requirements.

	CDR3α	CDR3β
TCRBuilder2+	3.48	3.15
Alphafold Multimer	2.80	2.30

SI Table 11: Mean number of conformation clusters per TCR sequence predicted, 1Å RMSD threshold.

Stage	Ensemble	Restrained Atoms	Restraint Strength (kJ mol^{-1} nm^{-2})	Duration	Start T (K)	End T (K)
1	Minimisation	Protein Heavy	4184.00	5000 steps	-	-
2	NVT	Protein Heavy	4184.00	0.2 ns	100	300
3	NPT	Protein Heavy	4184.00	0.2 ns	300	300
4	NPT	Protein Heavy	2092.00	0.5 ns	300	300
5	Minimisation	Backbone Heavy	2092.00	5000 steps	-	-
6	NPT	Backbone Heavy	2092.00	0.2 ns	300	300
7	NPT	Backbone Heavy	418.40	0.2 ns	300	300
8	NPT	Backbone Heavy	41.84	0.2 ns	300	300
9	NPT	-	-	1 ns	300	300

SI Table 12: Molecular dynamics equilibration protocol. Parameters and steps used during the equilibration of simulated systems.

2 SI Figures (1-22)

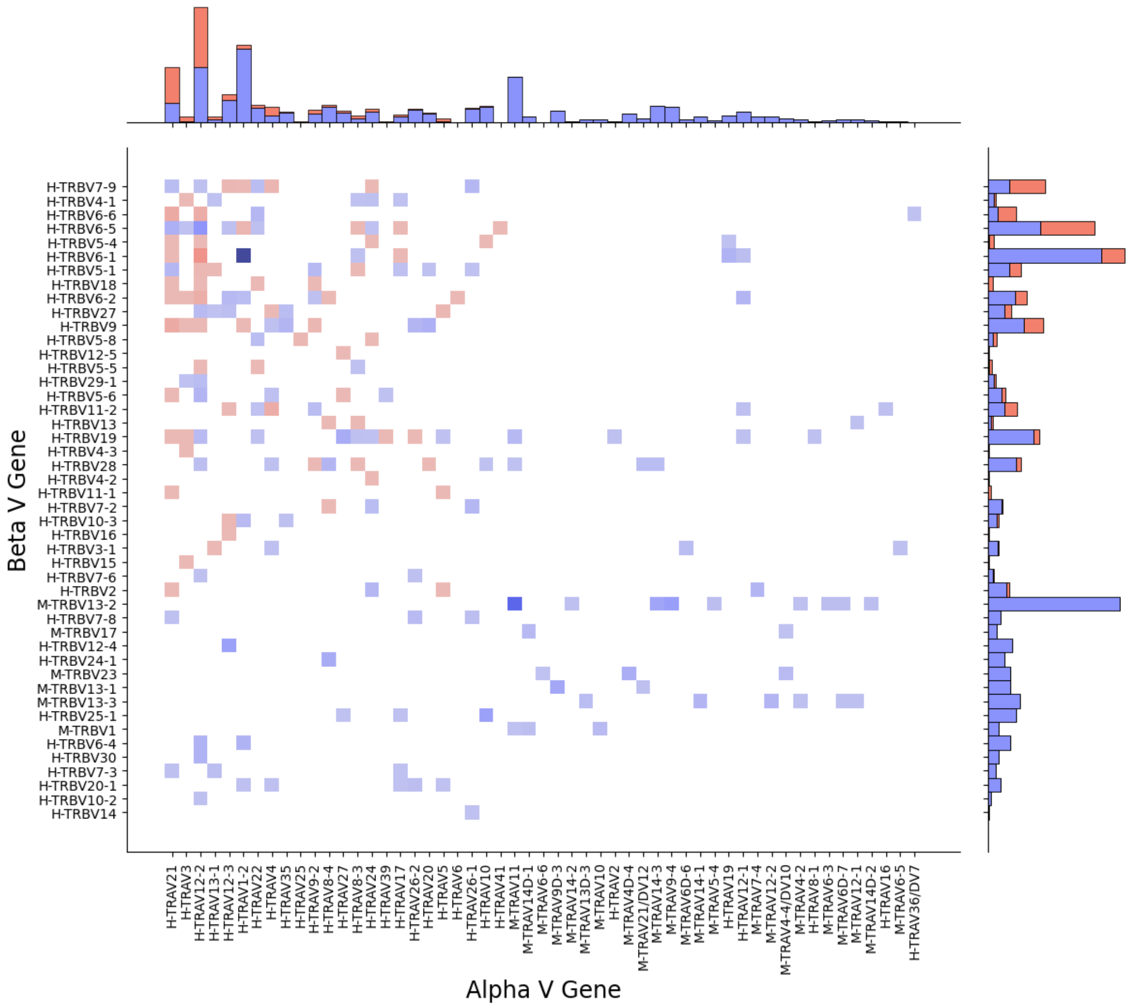

SI Figure 1: Joint distribution of TRAV and TRBV genes in the training data. STCRDab data in blue, Immunocore data in red. Human genes annotated 'H-', murine genes annotated 'M-'.

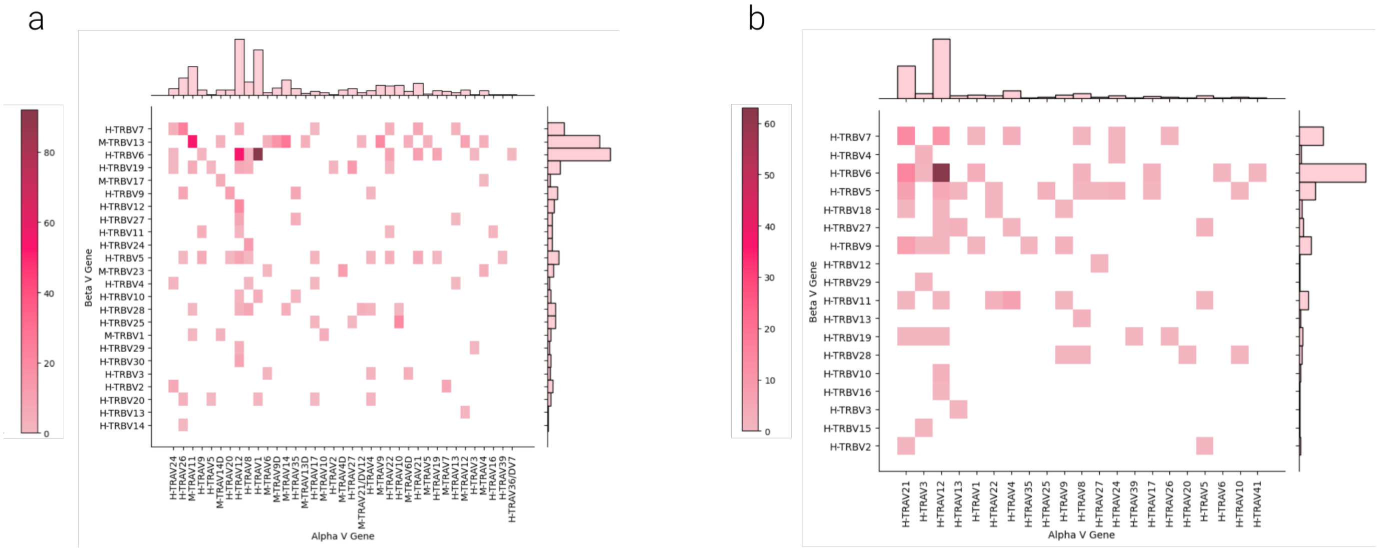

SI Figure 2: Joint distribution of alpha and beta V gene subgroups of TCR structures in the training data. a) Joint distribution of TCRs sourced from STCRDab. b) Joint distribution of TCRs provided by Immunocore. Genes beginning with 'H-' are human, those beginning with 'M-' are murine.

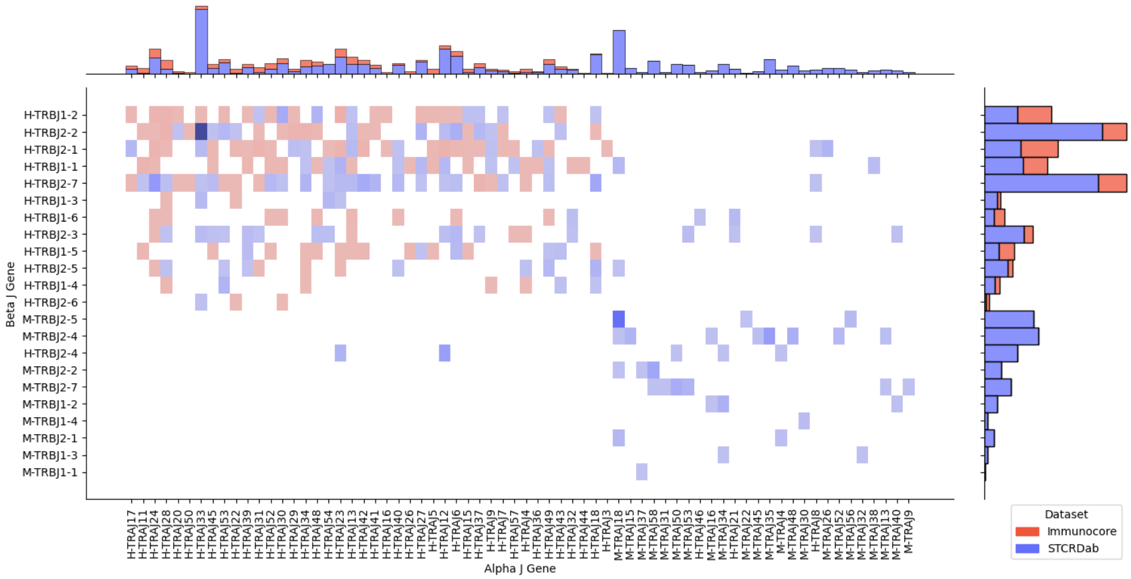

SI Figure 3: Joint distribution of alpha and beta J genes of TCR structures in the training data, coloured by data source (STCRDab in blue, Immunocore in red). Genes beginning with 'H-' are human, those beginning with 'M-' are murine.

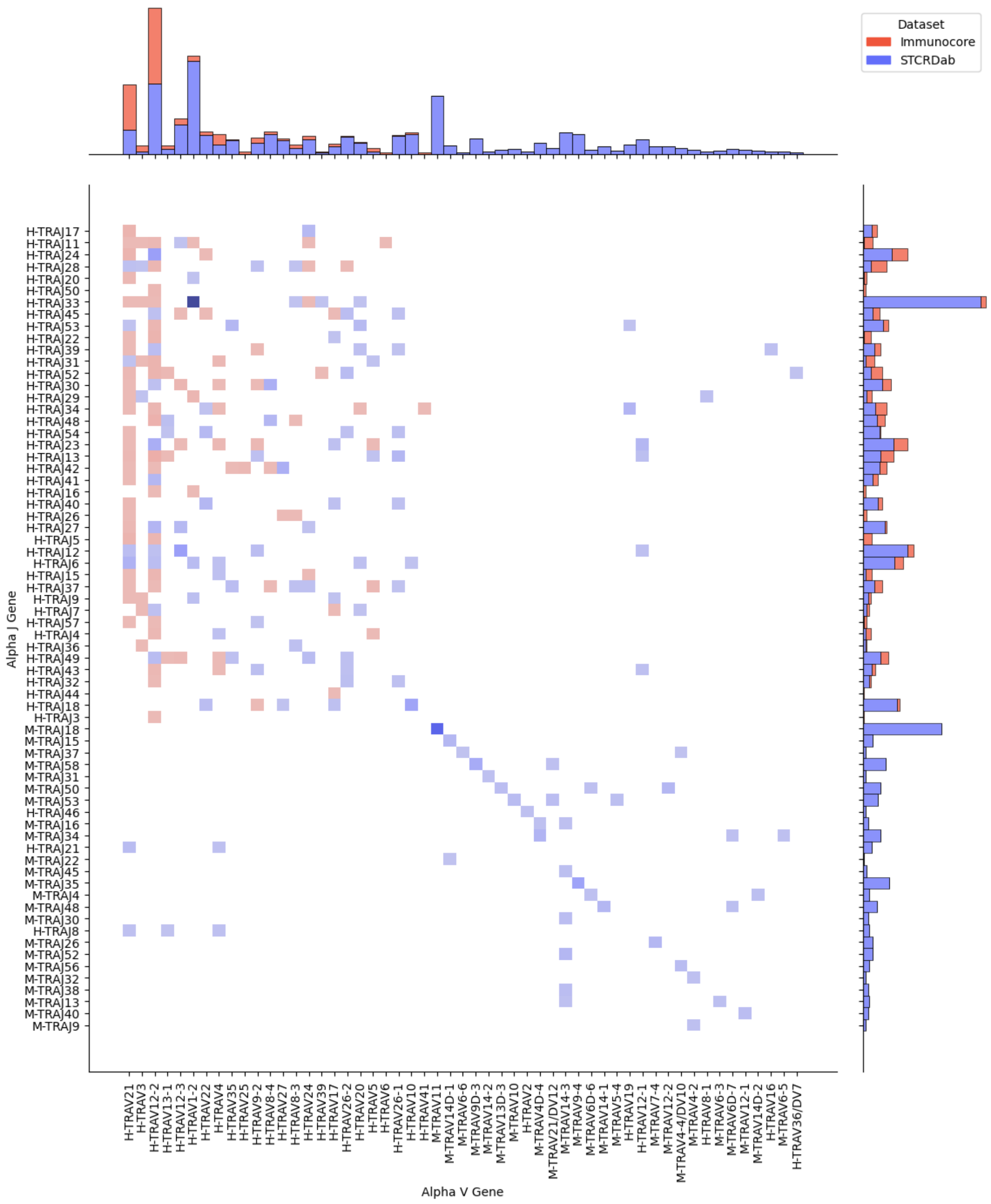

SI Figure 4: Joint distribution of TRAV and TRAJ genes of TCR structures in the training data, coloured by data source (STCRDab in blue, Immunocore in red). Genes beginning with 'H-' are human, those beginning with 'M-' are murine. The TRAV-TRAJ combinatorial space is visibly vaster and more sparsely sampled than the TRBV-TRBJ combinatorial space (SI Fig.5)

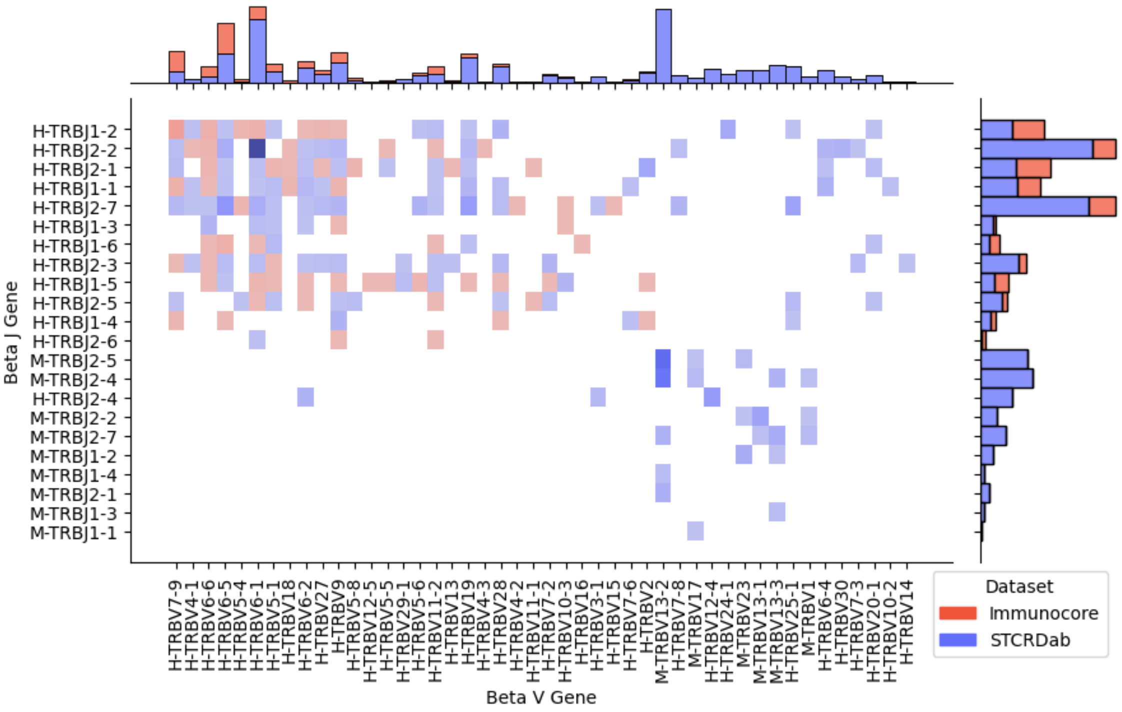

SI Figure 5: Joint distribution of TRBV and TRBJ genes of TCR structures in the training data, coloured by data source (STCRDab in blue, Immunocore in red). Genes beginning with ‘H-’ are human, those beginning with ‘M-’ are murine.

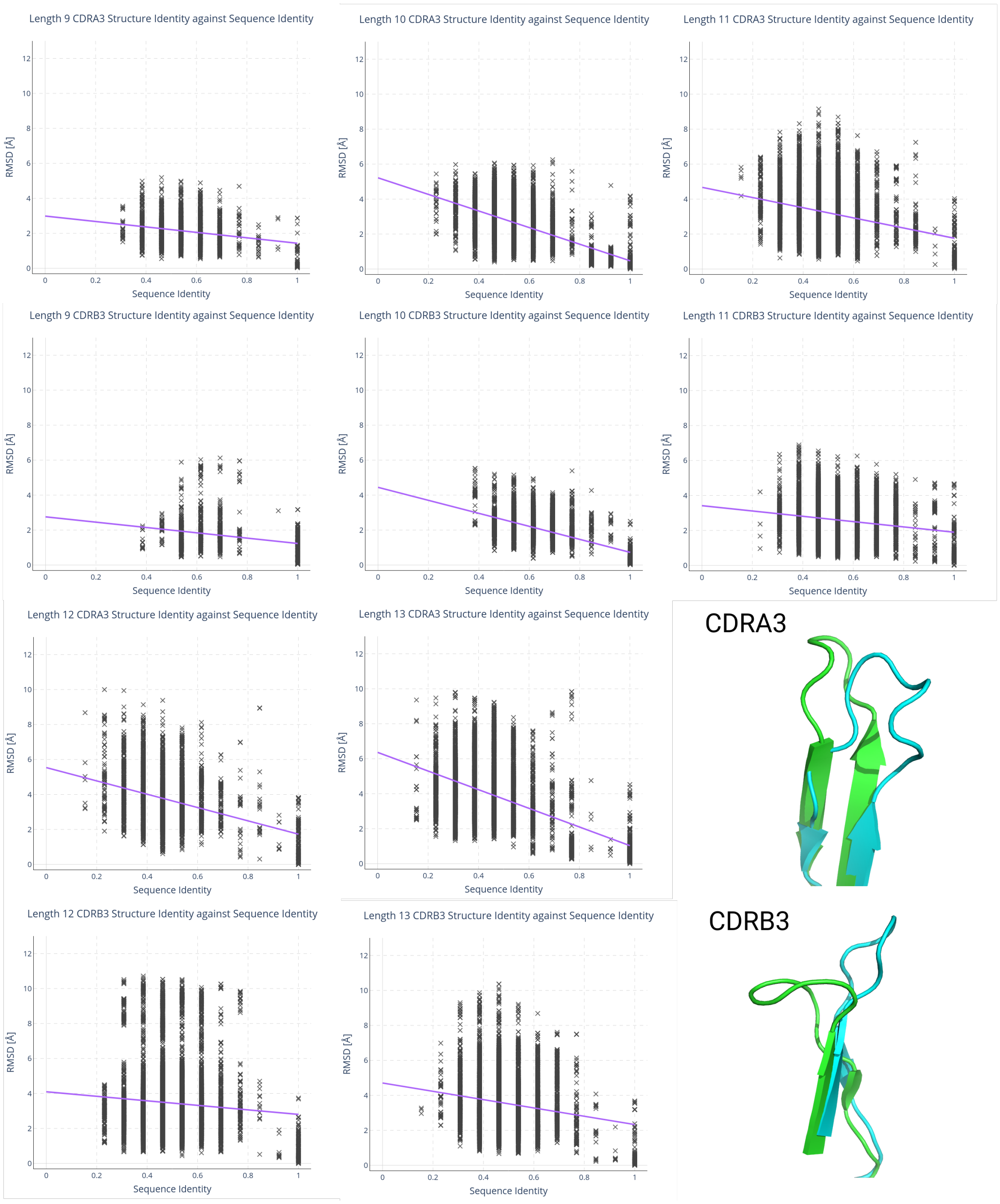

SI Figure 6: Pairwise RMSD *vs* sequence identity distribution of training data by loop length. Examples of identical sequence loops with high RMSD suggest that both CDR3α and CDR3β structures are diverse and are often not predictable from just the sequence of the CDR3 region.

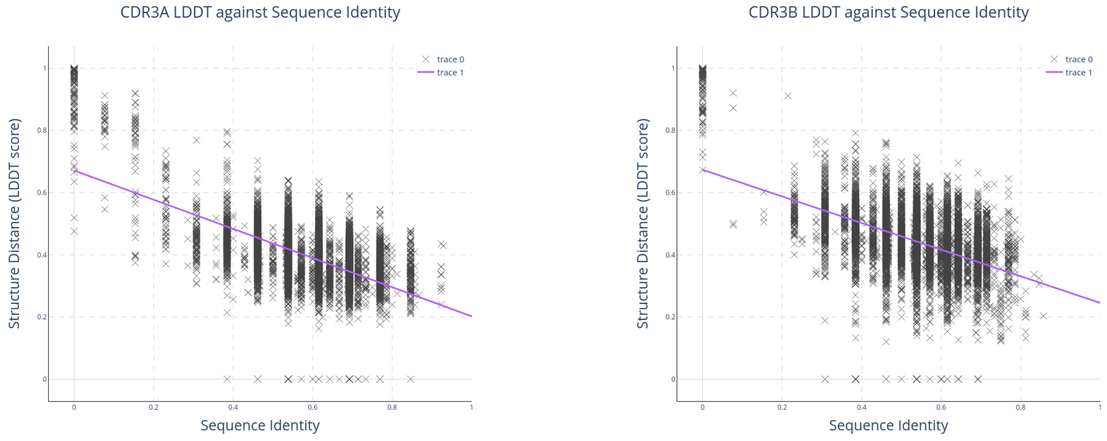

SI Figure 7: Pairwise LDDT score vs sequence identity

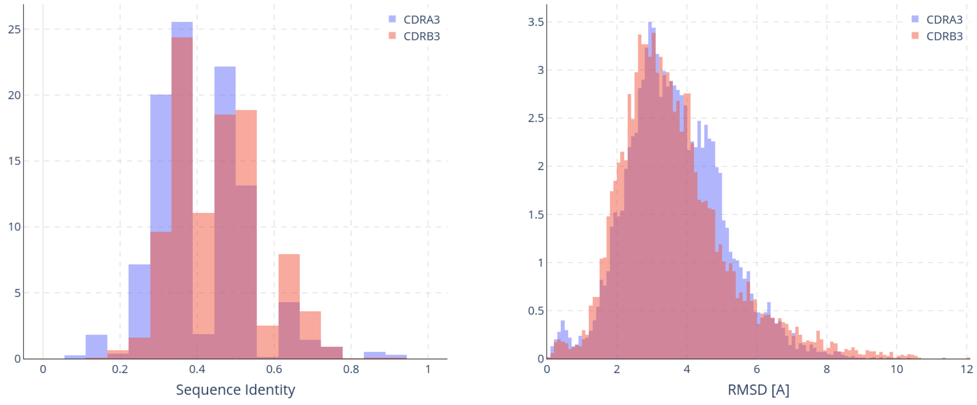

SI Figure 8: Pairwise sequence identity and RMSD (spline upsampled) distributions of the training data of TCRBuilder2+. The distributions of CDR3α and CDR3β suggest that CDR3α is as structurally/sequence diverse as CDR3β.

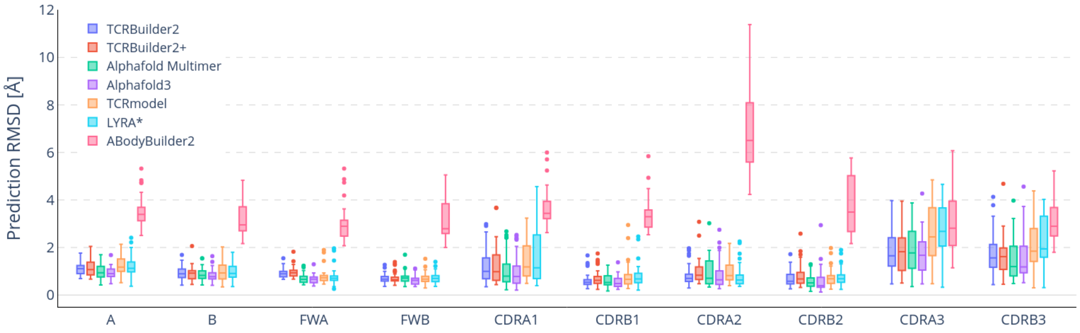

SI Figure 9: Comparison of prediction RMSD by different models, including only the 38/45 test structures for which the homology model LYRA [2] predicted a structure. Sample size of 38; boxes represent the inner quartiles, with the median value the dividing line; whiskers extend to points that lie within 1.5 times the interquartile range of the lower and upper quartile; observations beyond this range are displayed independently.

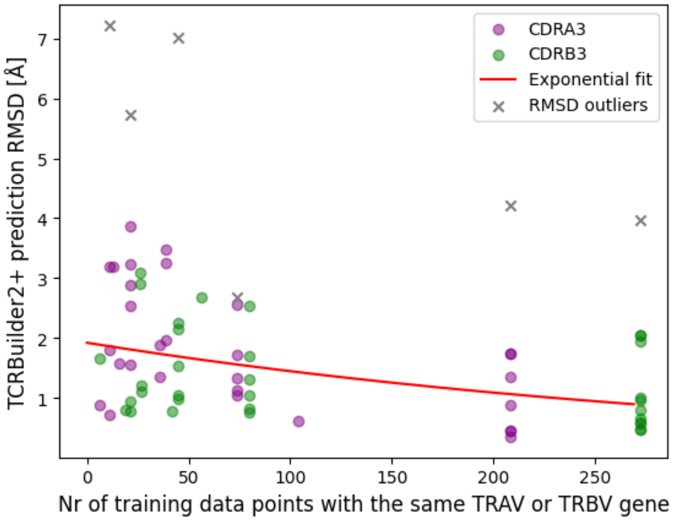

SI Figure 10: Test set TCRBuilder2+ prediction RMSD *vs.* variable gene abundance in training data for CDR3α (purple), and CDR3β (green). RMSD outliers of the test structures 7l1d, 7rk7 and 6zkx are shown in in grey. TCRBuilder2+ predictions improve for TCR sequences deriving from a variable gene observed more frequently in the training data. The data support a saturation effect; fitting an exponential curve suggests a slight decay, through the variation about the trend remains high.

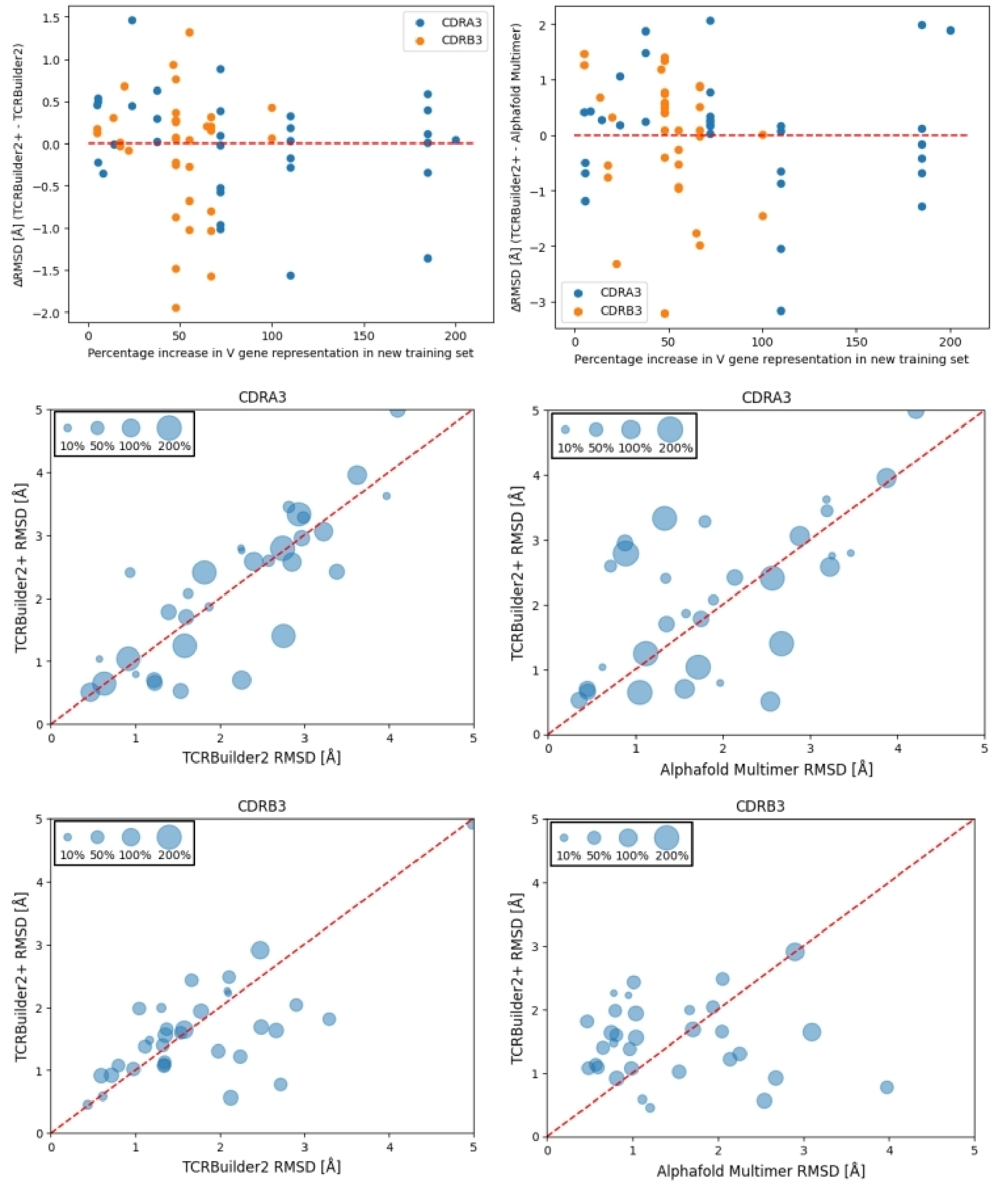

SI Figure 11: RMSD vs relative increase in V gene in training data. a) Difference in RMSD between TCRBuilder2 and TCRBuilder2+ (left) and Alphafold Multimer and TCRBuilder2+ (right) vs percentage increase in V gene in new training set (< 0Å means TCRBuilder2+ is better). b & c) CDR3α (b) and CDR3β (c) RMSD of TCRBuilder2+ vs TCRBuilder2 (left) and Alphafold Multimer (right). Bubble size indicates the percentage increase in datapoints for the TRBV gene of a given test sequence in the new training set compared to the old training set, red dashed line is the identity line at which models perform equivalently.

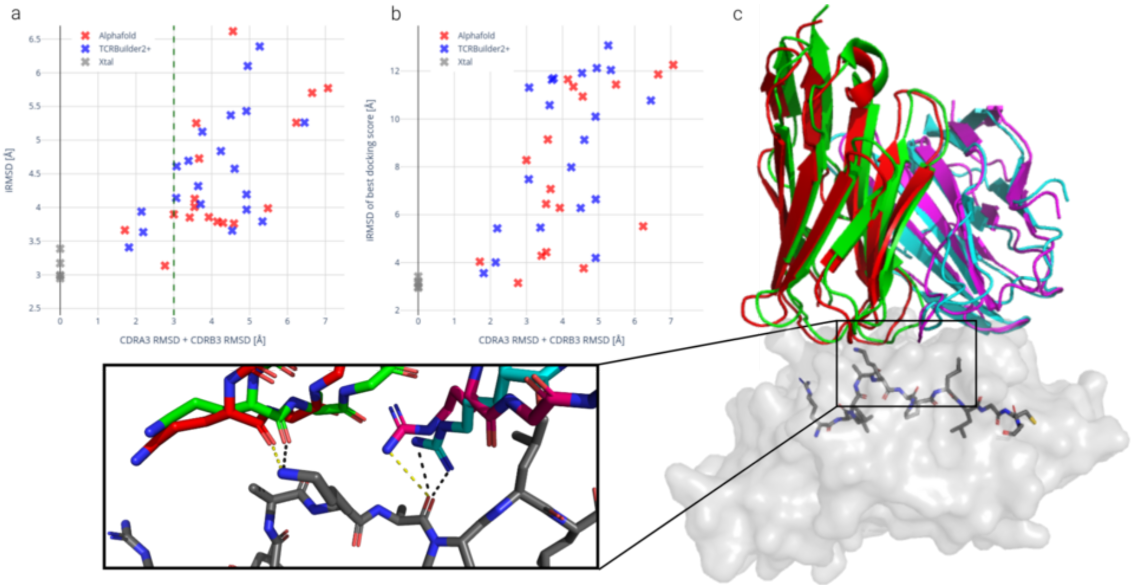

SI Figure 12: a) Best interface RMSD (iRMSD) of docked TCR structures to their cognate antigen. b) iRMSD of the best ranked dock by docking score. Crystal structure re-docks (grey) provide a baseline of realistically attainable iRMSD. Docking iRMSD clearly correlates with CDR3 loop prediction RMSD, with a joint RMSD <3 Å consistently retrieving a dock with iRMSD <4Å. Docking scores do not consistently retrieve the best docked score. c) Docked TCRBuilder2+ structure prediction of 6zkx to crystal structure pMHC. Predicted α and β chains are red and magenta respectively, original crystal structure α and β chains are green and cyan respectively. The pMHC is shown as a surface with the peptide as sticks. The docked CDR3α and CDR3β chains (zoom box) recapture the polar interactions with the peptide (docked interactions in yellow, crystal structure interaction in black).

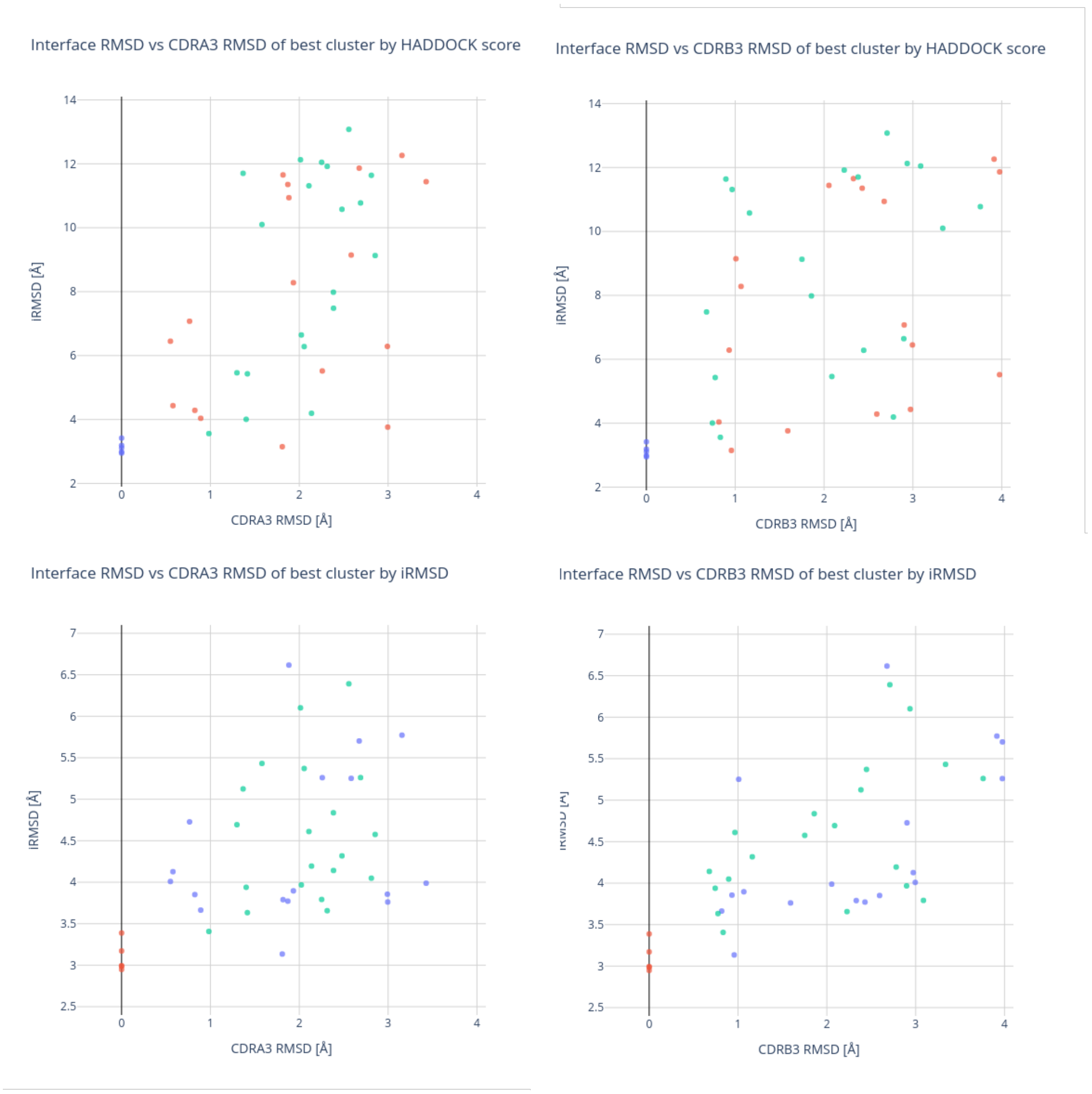

SI Figure 13: iRMSD of best clusters by iRMSD to crystal or HADDOCK score *vs.* CDR3 loop RMSD of TCRBuilder2+ and Alphafold Multimer predictions.

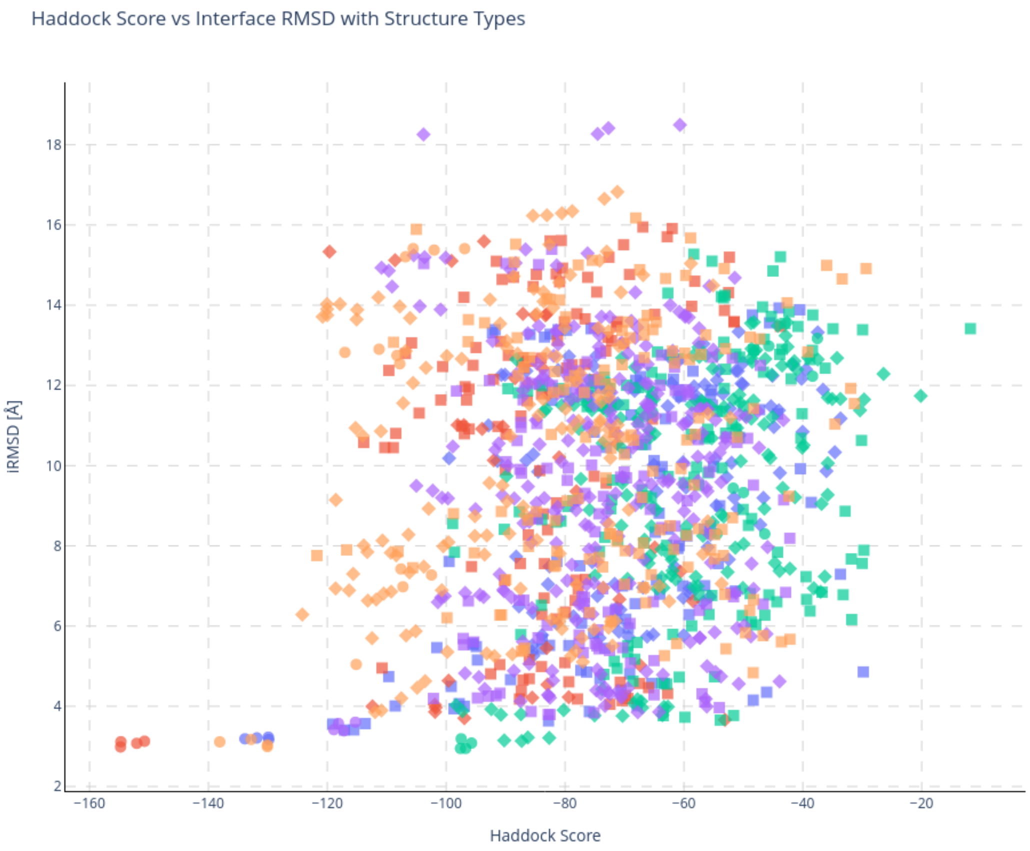

SI Figure 14: All docking clusters iRMSD *vs.* HADDOCK docking score, coloured by PDB code (6zkx: red, 7nme: blue, 7pb2: green, 7rm4: purple, 8d5q: orange). Shapes indicate the type of TCR structure (circle: crystal structure, square: TCRBuilder2+, diamond: Alphafold-Multimer).

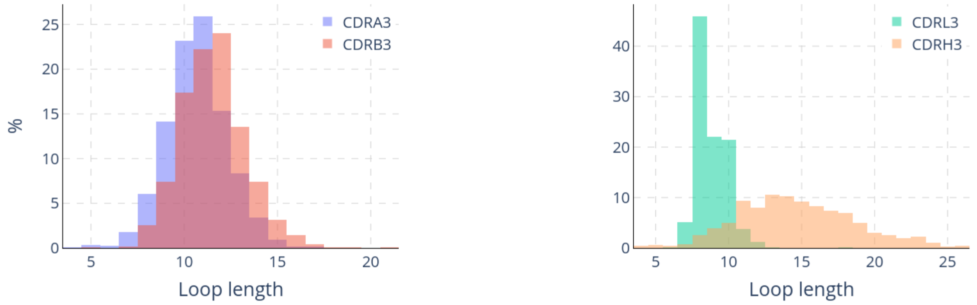

SI Figure 15: Distributions of CDR3 loop length of OTS sampled TCRs (left) and OAS sampled antibodies (right) accompanying Fig. 3c&d.

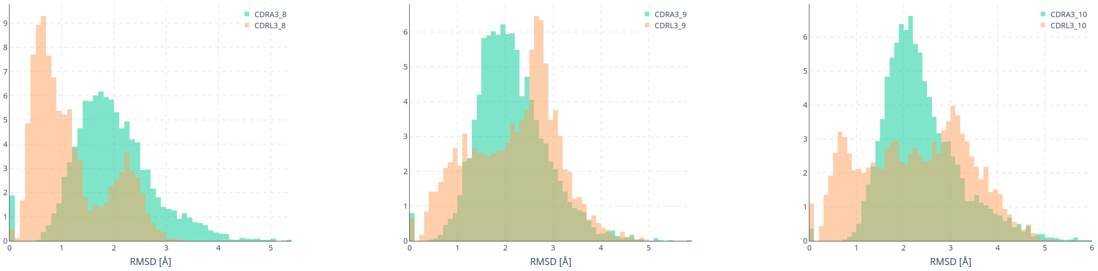

SI Figure 16: Length matched pairwise RMSD distributions of 1000 predicted TCR CDR3α and antibody CDRL3 loops predicted from OTS with TCRBuilder2+ and OAS with ABodyBuilder2 respectively. Loop lengths of 8, 9 and 10 residues from left to right respectively. CDRL3 RMSD histograms exhibit multiple modes, associated with canonical structures.

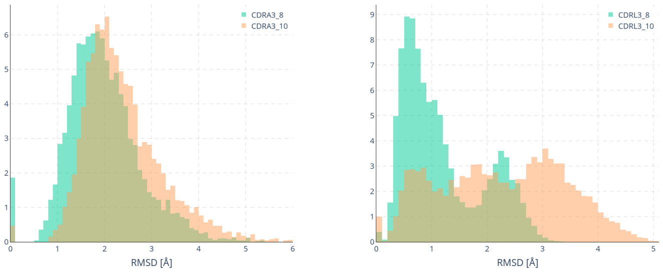

SI Figure 17: Length matched pairwise RMSD distributions of 1000 predicted TCR CDR3α and antibody CDRL3 loops predicted from OTS with TCRBuilder2+ and OAS with ABodyBuilder2 respectively. Comparison of RMSD distribution of loops of length 8 and 10. Mean RMSD increases with loop length.

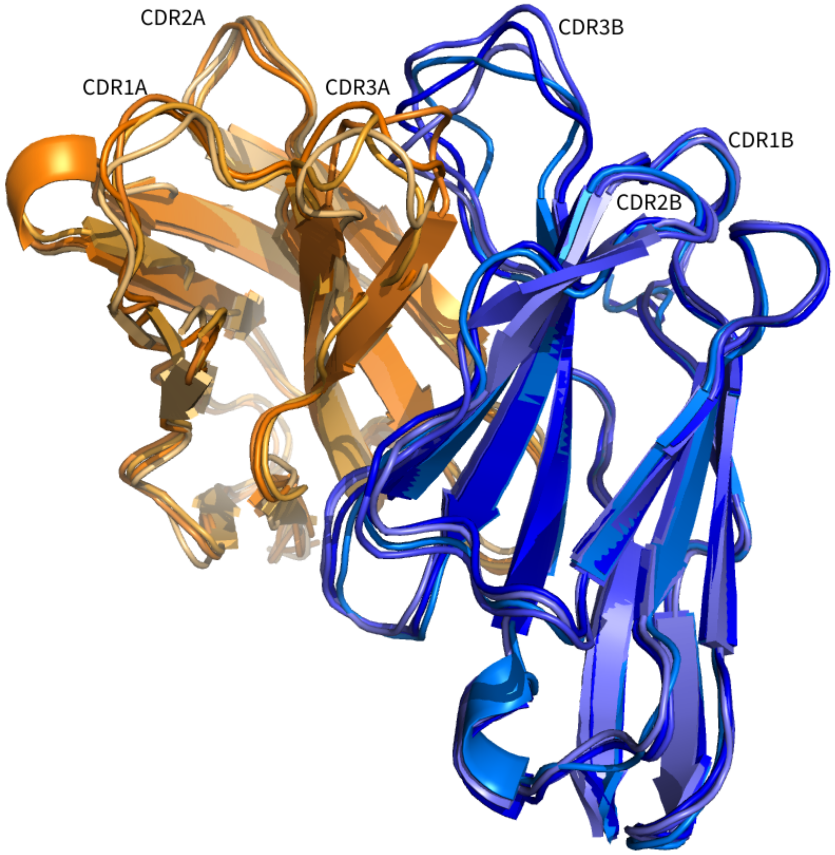

SI Figure 18: Example of TCRBuilder2+ ensemble of four predictions for test set TCR with PDB code 8GVB. Alpha chain in orange, beta chain in blue. The CDR3 lops occupy similar space but display more structural variation across predictions than the CDR1 and CDR2 loops of both chains.

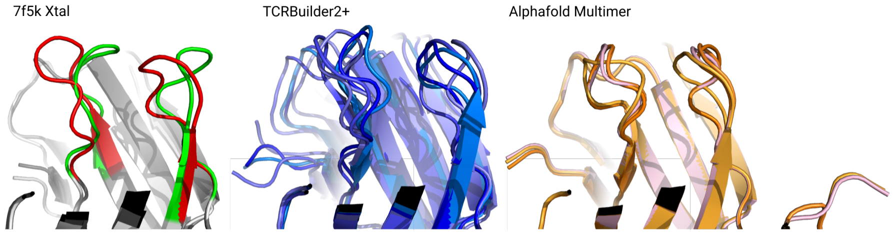

SI Figure 19: Starting conformations used in molecular dynamics (MD) simulations. The 7f5k crystal structure has two distinct conformations (red and green) for both the CDR3α and CDR3β loop. TCRBuilder2+ predicts four distinct conformations with which we initialised MD simulations. Alphafold Multimer makes 25 predictions, however many of them are structurally indistinguishable and cluster into a handful of predictions (see SI Fig. 20b). Here, we identified the predictions ranked 0, 1, 14, and 22 as structurally distinct and used them as starting structures for MD simulations.

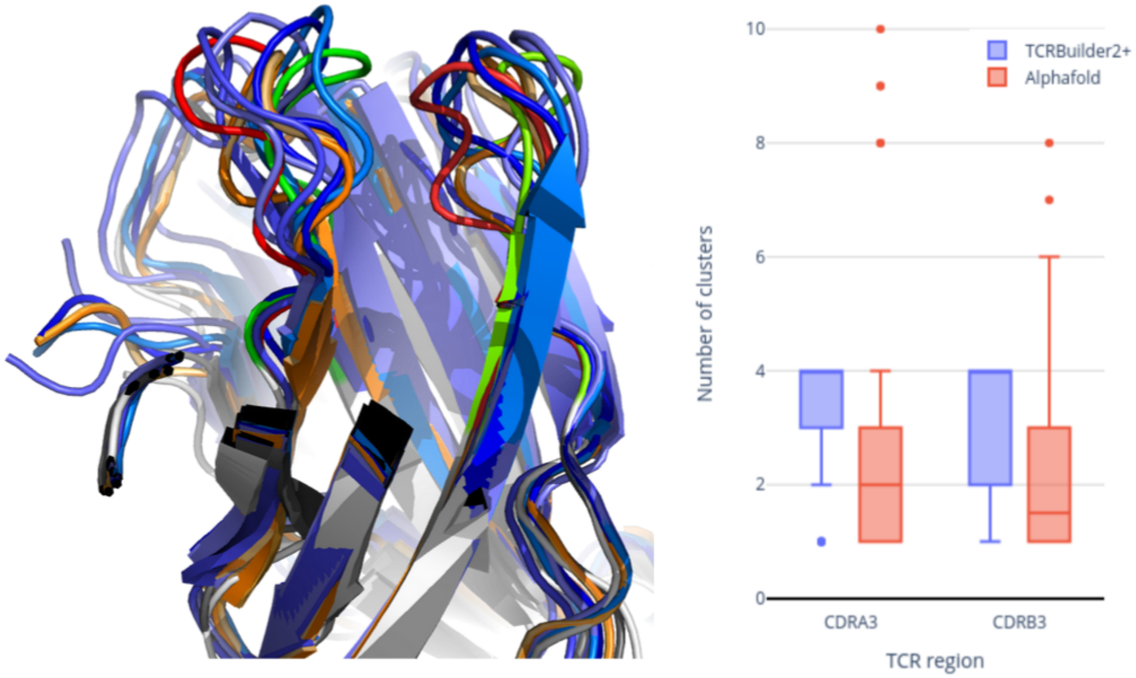

SI Figure 20: a) Multiple CDR3 conformations of 7f5k crystal structure and predicted structures in sliced view. CDR3α loops are on the left and CDR3β loops are on the right. The green and red loops are the two conformations observed in the apo TCR crystal structure. The blue loops are the four TCRBuilder2+ predictions, the orange loops are the top four ranked Alphafold-Multimer predictions. The top four Alphafold-Multimer predictions model the same loop, whereas TCRBuilder2+ produces distinct conformations. Neither TCRBuilder2+ nor Alphafold-Multimer predictions recapture either of the crystal structure conformations, but certain trends, for instance the 'inverted S' shape at the base of the CDR3α loop, are recapitulated. Molecular dynamics simulations suggest that the predicted conformations are physically plausible (SI Fig. 21) and lie within the space of commonly sampled backbone structure for CDR3 loops of this length. b) Number of distinct CDR3 loop structure clusters per sequence predicted for a cluster threshold of 1Å RMSD. Despite TCRBuilder2+ making four predictions, compared to Alphafold-Multimer's default of 25 predictions, the TCRBuilder2+ predictions typically fall into more clusters. Both models predict more conformations for CDR3α than CDR3β on average. Sample size of 45; boxes represent the inner quartiles, with the median value the dividing line; whiskers extend to points that lie within 1.5 times the interquartile range of the lower and upper quartile; observations beyond this range are displayed independently.

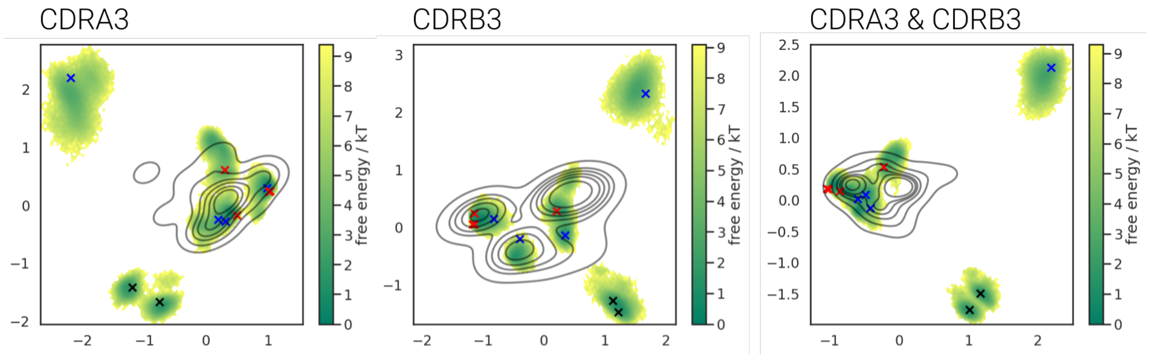

SI Figure 21: Time-lagged independent component analysis (tICA) projections in two dimensions of molecular dynamics trajectories of crystal and predicted CDR3 structures of 7f5k. The structures from which the simulations were initialised are marked as 'x'. The two crystal structure conformations are in black, the four TCRBuilder2+ predictions in blue, and the Alphafold-Multimer predictions in red. The green shading is the free energy distribution calculated from the states sampled in the MD trajectories. The black topography lines are the projected distribution of CDR3 structures of the same loop length as 7f5k. Predicted loop structures occupy spaces with nearby local free energy minima, indicating that the predicted conformations are relatively stable. TCRBuilder2+ samples a greater range of confomational space than Alphafold-Multimer, the crystal structure CDR3 loop structures lie beyond the modes of the background distribution.

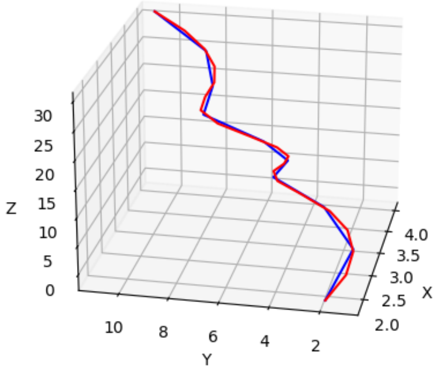

SI Figure 22: Demonstrative visualisation of spline up-sampling on 3D C_α coordinates of a simulated CDR loop. Original coordinates (blue) are up-sampled (red). The coordinates x, y and z are the atom positions in euclidian space.

3 SI Notes (1-3)

3.1 Accurate prediction of both CDR3α and CDR3β is required for TCR:pMHC interface characterisation using physics-driven docking

As presented in the main manuscript (Sec. 2.2), we sought to assess the extent to which accurate CDR3α or CDR3β loop structure predictions are key to obtaining useful putative binding poses. We used the software HADDOCK [3] to dock a subset of TCR structures from the test set, both crystal coordinates and models predicted by TCRBuilder2+ and Alphafold-Multimer, to crystal structures of their cognate antigens.

SI Fig. 12a shows the lowest interface RMSD (iRMSD) achieved across the docking run for each model alongside its CDR3 accuracy, while SI Fig. 12b compares CDR3 accuracy to the iRMSD of the 'best' dock defined by docking score. A visual inspection of the docks suggests that an iRMSD of less than 4 Å recaptures the pose with which the CDR3 loops bind to the antigen, with moderate recapitulation of the side-chain interactions (SI Fig. 12c).

The baseline re-docks of the crystal structures of TCRs suggest that the best attainable iRMSD is between 3 Å and 3.5 Å, due to the limited pose sampling of high-throughput docking (SI Fig. 12a & b). Meanwhile, the data on TCR models suggest clearly that accurate prediction of both the CDR3α and the CDR3β loops is required to enable accurate docking, defined as iRMSD < 4 Å, particularly when relying on docking score to retrieve the best poses. Specifically, we find that a total RMSD over the CDR3α and CDR3β < 3Å consistently enables the retrieval of a dock with an iRMSD < 4Å. It is not possible to identify a consistent accuracy threshold when considering the CDR3α or CDR3β individually (SI Fig. 13). This emphasises that both the CDR3α and CDR3β tend to contribute to antigen binding and aligns with our previous finding that CDR3α loops display similar structure diversity to CDR3β loops.

Though we were able to retrieve TCR:pMHC poses with iRMSD < 4 Å for nearly half of the TCRs, frequently these were not the docks with the best docking score. Quantitatively, while the docking scores of the best ranked dock correlate with iRMSD, only 31.1% (14/45) of the best docks by iRMSD also had the optimal docking score. Furthermore, as the docking scores are calculated as weighted sums of energy terms derived from the docked structure and vary across simulations it is not possible to determine a threshold for docking scores that holds for all TCRs and pMHCs

(SI Fig. 14). The unreliability of general pose ranking functions in this domain limits docking's practical applicability for investigating TCR:pMHC complementarity.

Finally, we investigated whether using multiple distinct TCR structure predictions for each sequence improves docking outcomes; TCRBuilder2+ generates four predictions per sequence (Fig. 1a) and Alphafold-Multimer generates 25, from which one is selected at run time as the best-ranked model. When we docked these alternate structure predictions, we find that the best-ranked TCRBuilder2+ model achieves the lowest iRMSD dock in three of five case studies, while this is only the case for one of five for Alphafold-Multimer. We hypothesise that this is due to the combinatorially expanded search space when including multiple conformations, thereby increasing the chances of retrieving a good docked pose. In the absence of a crystal structure, incorporating multiple distinct TCR conformation predictions in a pipeline may enable the retrieval of better docks, although we note again that the limitations of docking scores mean that identifying the best pose across runs remains challenging.

Molecular Docking Methods

We used HADDOCK [3] to run docking simulations. The active residues of the paratope were defined as residues of the six CDR loops according to IMGT numbering [4] and the active residues of the epitope were defined as the residues of the peptide presented by the pMHC. Unburied residues within 6.5Å of the peptide residues are defined as passive residues of the interaction site. No residues are defined as passive in the paratope.

We selected the TCR crystal structures in 6zkx, 7nme, 7pb2, 7rm4, and 8d5q for the docking simulations, as they are crystallised in complex with pMHC and their TCRBuilder2+ and Alphafold Multimer CDR3 prediction accuracies span a range of RMSD values. For each structure we ran docking simulations of the original crystal TCR structure and every TCR structure prediction by TCRBuilder2+ and Alphafold Multimer that clustered into different CDR3α or CDR3β conformations by greedy clustering with a threshold of 1Å. Each simulation results in 200 docked poses ranked with HADDOCK's docking score reflective of the enthalpy of the docked interface, clustered by RMSD to the best ranked dock.

We also evaluated docked pose quality by the interface RMSD (iRMSD) to the original crystal structure. iRMSD is calculated as the RMSD between all heavy atoms in the interface after aligning

the dock to the crystal by the MHC backbone atoms (N, C_α, C, and O). The interface was defined as all residues of the TCR and pMHC for which the C_α atoms were within 8Å.

3.2 Molecular dynamics suggests ensembles of models generated by TCR structure predictors may represent alternative, physically plausible apo states

TCR CDR3 loops have been hypothesised to be exhibit structural flexibility, particularly when compared to antibody CDR3 loops [5–9]. This provides a challenge to any method that offers a single TCR CDR3 loop conformation prediction and could disproportionately limit expected accuracy. In the main manuscript, we primarily benchmarked the 'top-ranked' predictions of TCRBuilder2+ and Alphafold-Multimer. However, these deep learning models can return multiple predicted structures, which we can analyse to assess whether they could represent physically plausible sets of conformations. TCRBuilder2+ is comprised of an ensemble of four models, each trained with different training and validation splits, and therefore produces four predictions for each sequence. Alphafold-Multimer uses an ensemble of five models which are fine-tuned in two stages from the best performing pre-trained model with different random seeds [10], each of which makes five different predictions for a single TCR sequence, resulting in 25 structures.

We began by investigating the conformational space sampled by the TCRBuilder2+ and Alphafold-Multimer predictions and find that TCRBuilder2+ explores more distinct conformations, despite Alphafold-Multimer producing over six-fold more models. We used greedy clustering with a threshold of 1Å RMSD to investigate the array of conformations predicted by TCRBuilder2+ and Alphafold Multimer (SI Fig. 20) and found that TCRBuilder2+ usually predicts four distinct conformations for both the CDR3α and CDR3β loop, while Alphafold Multimer predictions typically fall into just one or two clusters.

In line with our finding that CDR3α loops are at least as structurally diverse as CDR3β loops, both models predict a greater number of conformational clusters for the CDR3α than the CDR3β (SI Fig.20, SI Table 11). It is unfortunately impossible to disentangle whether this is due to greater uncertainty of the model predictions because the CDR3α is inherently more difficult to predict, hence leading to less conserved predictions by both models, or whether this is due to the CDR3α being intrinsically more flexible leading to the prediction of more diverse conformations. In the absence of experimental data that captures flexibility in TCR structures, we use molecular dynamics to assess the physical plausibility of the multiple predicted conformations, thereby evaluating whether predicted structures can be categorised as incorrect. Analysis of these simulations is consistent with

more than one CDR3 loop conformation being physically plausible, thereby both supporting the hypothesis that CDR3 loops of TCRs may be flexible [6, 7, 9, 11], and questioning whether regressing structure prediction models towards single crystal structures leads to useful *in-vivo* structure predictions.

Due to the computational expense of running molecular dynamics simulations, we examined the case study of the *apo* crystal structure 7f5k, which we select on the basis that it is not in complex with an antigen, is contained in our filtered test set (SI Table 1), and exhibits two distinct conformations for both the CDR3α and CDR3β in the crystal structure(SI Fig. 20).

We then selected four predictions with distinct conformations made by both TCRBuilder2+ and Alphafold-Multimer. Since Alphafold-Multimer makes 25 predictions, we use the four structures that ranked best by pTM score and fall into different conformation clusters. This results in a final set of 10 structures for the same TCR sequence with which we initialise molecular dynamics simulations (SI Fig. 19).

SI Fig. 21 shows the free energy landscape sampled by the simulation trajectories. After being initialised from their predicted or crystal conformation (SI Fig. 19), the CDR3 loops explore the local conformation space, but no macro transitions away from the initial predictions are observed. This suggests that while the predictions do not recapture the loop structure of the crystallised TCR, the predicted structures are energetically stable within the MD forcefield. This supports the hypothesis that multiple loop conformations may be valid, particularly for apo (unbound) TCRs, and that, despite neither model being trained to predict multiple conformations, both Alphafold-Multimer and TCRBuilder2+ could be capturing multiple plausible structures.

Molecular dynamics methods

We performed unbiased molecular dynamics simulations on the TCR in PDB entry 7f5k, as it displays multiple distinct conformations for both the CDR3α and CDR3β in the crystal structure and was not included in the training set. MD was run both on the crystal poses and on models of the TCR sequence generated by TCRBuilder2+ and AlphaFold-Multimer,

All systems were prepared, and simulations performed, using OpenMM v7.7 [12]. N-methyl groups were used to cap C-termini using an in-house script. Next, using pdbfixer [12], we protonated the models at a pH of 7.5, soaked them in truncated octahedral water boxes with a padding distance

of 1 nm, and added sodium or chloride counter-ions to neutralise charges and then NaCl to an ionic strength of 150 nM. Prior to placing molecules in boxes, we aligned all variable region structures on the first copy of the variable region in the 7f5k crystal structure. We parameterised the systems using the Amber14-SB forcefield [13] and modelled water molecules using the TIP3P-FB model [14]. Non-bonded interactions were calculated using the particle mesh Ewald method [15] using a cut-off of distance of 0.9 nm, with an error tolerance of 5×10^{-4}. Water molecules and heavy atom-hydrogen bonds were rigidified using the SETTLE [16] and SHAKE [17] algorithms, respectively. We used hydrogen mass repartitioning [18] to allow for 4 fs time steps. Simulations were run using the mixed-precision CUDA platform in OpenMM using the Middle Langevin Integrator with a friction coefficient of 1 ps^{-1} and the Monte-Carlo Barostat set to 1 atm. We equilibrated systems using a multi-step protocol detailed in SI Table 12. Following equilibration, we performed 2 µs of unrestrained simulation of the NPT ensemble at 300 K.

We performed dimensionality reduction on the resultant trajectories through Time-lagged independent component analysis (TICA) [19] using PyEMMA2 [20]. We selected the sine and cosine transforms of either CDR3α, CDR3β, or CDR3α and CDR3β loop backbone dihedral angles as features, and set 5 ns as the lag time. We also embedded the starting structures for each simulation in the same resultant space. Lastly, to compare other known experimental structures to the conformations observed during simulations, we identified structures of TCR receptors in our training set where both the CDR3α and CDR3β loops had the same number of residues as the simulated system (11 residues according to the IMGT definition [4] for either loop) and embedded their CDR3 backbone dihedrals in the same coordinate space.

3.3 Structural distance calculation for length-mismatched CDR loops

Protein structures are typically compared by calculating the RMSD between atom positions, often restricted to C_α. However, this poses a problem when comparing chains of different lengths. In order to compare different length CDR3 loop structures, we implement a method for upsampling of the backbone coordinates to match in length. We use spline upsampling to re-sample the shorter loop C_α coordinates by polynomial interpolation, and compare the new coordinates. For chains of the same length this distance calculation is equivalent to standard RMSD. Empirically, we find that this method is upper bound by the RMSD, which we find by calculating the distance between two equivalent length loops after upsampling. SI Fig. 22 is an illustrative example of original backbone coordinates (blue) and the upsampled coordinates (red). We also implemented a version of the dynamic time warping (DTW) algorithm, an alternative method based on optimally matching coordinates in the longer loop to coordinates in the shorter loop. While this method is in use, it is liable to spurious, non-RMSD like results when comparing very different structures, such that sequence aligned coordinates are substantially further than DTW match coordinates, resulting in low distances despite structural dissimilarity. We therefore opt for the former method; both methods are implemented as a small python package with a common API (`https://github.com/npqst/different-length-euclidian-rmsd`).

SI References

[1] V. Giudicelli. “IMGT/GENE-DB: a comprehensive database for human and mouse immunoglobulin and T cell receptor genes”. In: *Nucleic Acids Research* (2004). DOI: 10.1093/nar/gki010.

[2] Michael Schantz Klausen et al. “LYRA, a webserver for lymphocyte receptor structural modeling”. In: *Nucleic Acids Research* (2015). DOI: 10.1093/NAR/GKV535.

[3] G. C.P. Van Zundert et al. “The HADDOCK2.2 Web Server: User-Friendly Integrative Modeling of Biomolecular Complexes”. In: *Journal of Molecular Biology* (2016). DOI: 10.1016/j.jmb.2015.09.014.

[4] Marie Paule Lefranc et al. “IMGT, the international ImMunoGeneTics information system®”. In: *Nucleic Acids Research* (2005). DOI: 10.1093/nar/gki065.

[5] Wing Ki Wong, Jinwoo Leem, and Jinwoo Leem. “Comparative Analysis of the CDR Loops of Antigen Receptors”. In: *Frontiers in Immunology* (2019). DOI: 10.3389/FIMMU.2019.02454/BIBTEX.

[6] Christopher J. Holland et al. “In Silico and Structural Analyses Demonstrate That Intrinsic Protein Motions Guide T Cell Receptor Complementarity Determining Region Loop Flexibility”. In: *Frontiers in Immunology* (2018). DOI: 10.3389/fimmu.2018.00674.

[7] James E. Crooks et al. “The Hypervariable Loops of Free TCRs Sample Multiple Distinct Metastable Conformations in Solution”. In: *Frontiers in Molecular Biosciences* (2018). DOI: 10.3389/fmolb.2018.00095.

[8] Arjun K. Mishra and Roy A. Mariuzza. “Insights into the Structural Basis of Antibody Affinity Maturation from Next-Generation Sequencing”. In: *Frontiers in Immunology* (2018). DOI: 10.3389/fimmu.2018.00117.

[9] Brian M. Baker et al. “Structural and dynamic control of T-cell receptor specificity, cross-reactivity, and binding mechanism”. In: *Immunological Reviews* (2012). DOI: 10.1111/j.1600-065X.2012.01165.x.

[10] Richard Evans et al. “Protein complex prediction with AlphaFold-Multimer”. In: *bioRxiv* (2022). DOI: 10.1101/2021.10.04.463034.

[11] Stephanie Gras et al. “A structural voyage toward an understanding of the MHC-I-restricted immune response: Lessons learned and much to be learned”. In: *Immunological Reviews* (2012). DOI: 10.1111/j.1600-065X.2012.01159.x.

[12] Peter Eastman et al. “OpenMM 7: Rapid development of high performance algorithms for molecular dynamics”. In: *PLoS Computational Biology* (2017). DOI: 10.1371/journal.pcbi.1005659.

[13] James A. Maier et al. “ff14SB: Improving the Accuracy of Protein Side Chain and Backbone Parameters from ff99SB”. In: *Journal of Chemical Theory and Computation* (2015). DOI: 10.1021/acs.jctc.5b00255.

[14] W.L.; Jorgsen et al. “Comparison of simple potential functions for simulating liquid water”. In: *Water in Organic Synthesis* (1983). DOI: 10.1055/sos-sd-206-00006.

[15] Tom Darden, Darrin York, and Lee Pedersen. “Particle mesh Ewald: An N·log(N) method for Ewald sums in large systems”. In: *The Journal of Chemical Physics* (1993). DOI: 10.1063/1.464397.

[16] Shuichi Miyamoto and Peter A Kollman. “Settle: An analytical version of the SHAKE and RATTLE algorithm for rigid water models”. In: *Journal of Computational Chemistry* (1992). DOI: 10.1002/jcc.540130805.

[17] Jean Paul Ryckaert, Giovanni Ciccotti, and Herman J C Berendsen. “Numerical integration of the cartesian equations of motion of a system with constraints: molecular dynamics of n-alkanes”. In: *Journal of Computational Physics* (1977). DOI: 10.1016/0021-9991(77)90098-5.

[18] Chad W Hopkins et al. “Long-time-step molecular dynamics through hydrogen mass repartitioning”. In: *Journal of Chemical Theory and Computation* (2015). DOI: 10.1021/ct5010406.

[19] Lutz Molgedey and H G Schuster. “Separation of a mixture of independent signals using time delayed correlations.” In: *Physical review letters* (1994).

[20] Martin K. Scherer et al. “PyEMMA 2: A Software Package for Estimation, Validation, and Analysis of Markov Models”. In: *Journal of Chemical Theory and Computation* (2015). DOI: 10.1021/acs.jctc.5b00743.
