# Supplementary material for: T-cell receptor structures and predictive models reveal comparable alpha and beta chain structural diversity despite differing genetic complexity
Source: Commun Biol. 2025 Mar 4;8:362. doi: 10.1038/s42003-025-07708-6 (PMC11880327; doi:10.1038/s42003-025-07708-6)
Supplement: Supplementary file 2 — Description of Additional Supplementary Files [file 42003_2025_7708_MOESM2_ESM.docx]

Description of Additional Supplementary Files

**File name:** Supplementary Data 1

**Description:** Supplementary data for reproducibility of figures and results.

**File name:** Supplementary Data 2

**Description:** Supplementary data containing RMSD distance matrices for analysis and results presented in Figure 3.

**File name:** Supplementary Data 3

**Description:** Supplementary data containing sequence identity distance matrices for analysis and results presented in Figure 4.
